# Contribution of the seminal microbiome to paternal programming

**DOI:** 10.1093/biolre/ioae068

**Published:** 2024-05-02

**Authors:** Justine Kilama, Carl R Dahlen, Lawrence P Reynolds, Samat Amat

**Affiliations:** Department of Microbiological Sciences, North Dakota State University, NDSU Department 7520, Fargo, ND 58108-6050, USA; Department of Animal Sciences, and Center for Nutrition and Pregnancy, North Dakota State University, NDSU Department 7630, Fargo, ND 58108-6050, USA; Department of Animal Sciences, and Center for Nutrition and Pregnancy, North Dakota State University, NDSU Department 7630, Fargo, ND 58108-6050, USA; Department of Microbiological Sciences, North Dakota State University, NDSU Department 7520, Fargo, ND 58108-6050, USA

**Keywords:** seminal microbiome, paternal programming, fetal programing, offspring development and health

## Abstract

The field of Developmental Origins of Health and Disease has primarily focused on maternal programming of offspring health. However, emerging evidence suggests that paternal factors, including the seminal microbiome, could potentially play important roles in shaping the developmental trajectory and long-term offspring health outcomes. Historically, the microbes present in the semen were regarded as inherently pathogenic agents. However, this dogma has recently been challenged by the discovery of a diverse commensal microbial community within the semen of healthy males. In addition, recent studies suggest that the transmission of semen-associated microbes into the female reproductive tract during mating has potentials to not only influence female fertility and embryo development but could also contribute to paternal programming in the offspring. In this review, we summarize the current knowledge on the seminal microbiota in both humans and animals followed by discussing their potential involvement in paternal programming of offspring health. We also propose and discuss potential mechanisms through which paternal influences are transmitted to offspring via the seminal microbiome. Overall, this review provides insights into the seminal microbiome-based paternal programing, which will expand our understanding of the potential paternal programming mechanisms which are currently focused primarily on the epigenetic modifications, oxidative stresses, and cytokines.

## Teaser

This review explores the potential role of seminal microbiota in transferring paternal programming to offspring and proposes possible mechanisms of transmission, thus expanding the current knowledge of Developmental Origins of Health and Disease.

## Introduction

The concept of developmental programming, or Developmental Origins Of Health and Disease (DOHaD), refers to the notion that the environmental influences of the parents (including malnutrition, overnutrition, stress, smoking andtoxins, etc.) before or during conception and throughout gestation potentially results into enduring physiological, metabolic, and epigenetic changes in the offspring, which ultimately increase their susceptibility to disease and poor growth development in adulthood [1–3]. Many studies have shown that maternal health and nutrition during pregnancy and lactation can have a significant influence on their offspring health. Epidemiological studies of pregnant women who faced severe hunger during pregnancy because of food shortages during war-time, also known as the “Dutch Hunger Winter” or the “Dutch Famine,” provided foundational evidence of developmental programming [4]. It was reported that regardless of birth weight, children born during the famine had increased susceptibility to chronic diseases, altered organ function, and higher mortality [5]. Additional studies also showed a higher cardiovascular disease–related mortality in males with low birthweights, demonstrating that developmental programming effects are influenced by both gestational stage of exposure and offspring sex [6]. Since then, several progressive investigations performed using animal models illustrated the impacts of maternal diet on multiple offspring organ systems such as cardiovascular, musculoskeletal, digestive, reproductive, immunological, endocrine, and central nervous systems [1, 7–10].

However, as our understanding of the DOHaD mediated by the maternal side continues to expand, the involvement of paternal exposome in developmental programming of offspring health and disease has also recently begun to be appreciated [11–15]. Paternal programming encapsulates the idea that pre-mating paternal factors have the capacity to modify the semen, thereby exerting a substantial influence on the developmental trajectory and long-term health of the offspring [1, 16]. Various factors including paternal exposure to environmental toxicants, dietary stress, as well as various lifestyle and health status prior to conception have been reported to influence fetal programming and offspring health [17, 18].

The potential mechanisms through which paternal effects are transmitted to the offspring have been suggested to be associated with epigenetic modifications, cytokine profiles, oxidative stresses, and DNA damage in sperm cells, as well as semen components including the seminal microbiota [1, 19, 20]. Recent sequencing-based studies have revealed that there is a diverse and dynamic commensal microbiota present in the semen of humans [21, 22], bovine bulls [23–25], and horse stallions [26]. Moreover, emerging reports suggest that microbes associated with the semen could “hitchhike” to the vagina during coitus and interact with vaginal microbes to form so called “complementary semino-vaginal microbiome” [27]. This phenomenon highlights the potential roles that the seminal microbiota may play not only in influencing fertility outcomes but also in transferring paternal effects to the offspring [25, 28]. Thus, in this current review, we first briefly introduce the concept of paternal programming followed by a summary of the current stage of knowledge on the seminal microbiota in human and animal species. We then discuss the potential involvement of the seminal microbiome in paternal programming of offspring health. In addition, we propose and discuss the seminal microbiome as one of the potential mechanisms mediating the transfer of paternal influences to the offspring. Given the recent increase in research efforts towards the modulation of the maternal microbiome to enhance offspring health, understanding the contribution of the seminal microbiome to paternal programming is important in directing future research to harness the paternal microbiome for improved offspring health.

## Paternal programming and its associated mechanisms

### Paternal programming

With the accumulation of knowledge on the DOHaD, recent studies have revealed more complex layers of interactions among the genome, the environment, and epigenetics in shaping the offspring health. As our understanding deepens, the acknowledgment of multiple parental factors that can influence offspring health outcomes has been extended to include the contribution of paternal factors in developmental programming [11]. The perspective of paternal programming is centered on the notion that the fathers’ pre-conception environment has an impact on the seminal plasma and sperm cells, which can then impact on the embryonic developmental trajectory as well as the long-term metabolic and reproductive fitness of the offspring [1, 16]. This concept of paternal programming, which aims to uncover the paternal influence on offspring health within the framework of the DOHaD hypothesis is gaining recognition in both animal and human research [14, 29, 30]. Some authors have gone as far as suggesting that this emerging concept should be officially termed the “Paternal Origins of Health and Disease” paradigm (POHaD) [16, 29, 30] while others are proposing that it should simply be termed “Origins of Health and Disease (OHaD)” to accommodate maternal, paternal, and siblings Origins of Health and Disease.

### Evidence on paternal programming from rodent animal models

A growing body of research from rodent models is shedding light on potential mechanisms through which paternal factors including dietary predilections [14, 31], exposure to environmental stressors like toxicants [32], and epigenetic modifications within spermatozoa [33] can have long-term effects on offspring health. A summary of evidence from multiple studies illustrating the paternal programming effects in mice and livestock species is presented in Table 1. For instance, plethora of studies using mainly rodent animal-based models have identified links between paternal diets, obesity, low sperm quality and increased risks of cardio-metabolic disorders (e.g. obesity and type 2 diabetes) in offspring [34–37]. For example, poor paternal diets like a low-protein diet [15] or over/under nutrition [12] during spermatogenesis had paternal programming impacts on the postnatal metabolism and physiology of the offspring mice. Recently, Claycombe-Larson and colleagues investigated the influence of paternal high-fat (HF) diet and exercise on the risk of type 2 diabetes in offspring using mice model (3-week-old male C57BL/6 mice [F1]; [31, 38]. They observed that the paternal (F0) HF diet reduced placental and fetal tissue weights as well as nutrient transporter gene expression in the placental tissues of male offsprings (F1). However, paternal exercise (voluntary wheel running) for 3 months before mating increased mouse placental tissue weight, enhanced placental nutrient transporter gene expression, and increased fetal weights but reduced placental inflammatory gene expression [38]. Bromfield and colleagues investigated the implications of surgically excising the seminal vesicle glands in mice in order to remove the plasma proportion of seminal fluid [39]. They observed a poor conception and late gestational placental hypertrophy during pregnancy in females mice mated by males with excised vesicular glands. Alteration in patterns of growth and metabolic indicators, like obesity and hormone imbalances, were observed in the offspring, especially in male pups. The authors argued that the sperm injury and insufficient seminal fluid in the female tract were the causes of these consequences [39]. In addition, adult male progeny exhibited heightened adiposity despite transferring normal embryos to females mated with males that lacked seminal vesicle gland. Moreover, the absence of seminal plasma also reduced the expression of embryotrophic *Lif, Csf2, Il6,* and *Egf* but increased the level of apoptosis-inducing factors *trail* in the oviduct which could have profound impacts during preimplantation embryonic development [39]. They also observed that embryos formed in female reproductive tracts that were not exposed to the seminal plasma were aberrant from the early cleavage phases, but these abnormalities were partially ameliorated upon culturing *in vitro* [39]. These results demonstrated that the composition of the paternal seminal fluid influences the fetal development [40] and health of his male offspring. This study also showed that the seminal fluid’s influence on the periconceptional environment extends beyond sperm protection to include indirect effects on preimplantation embryos through regulating the expression of embryotrophic inflammatory mediators in the oviduct.

**Table 1:** Summarized evidence from several studies illustrating paternal programming effects in different mice and livestock species

Species	Experiment conducted	Links to paternal programming	Citation
C57BL/6 mice	Mice were fed a Western-style diet (WD) with 4.1 kcal/g, 46.1% carbohydrate, 15.3% protein, 17.9% fat vs. a Control diet with 2.7 kcal/g, 59.9% carbohydrate, 16.1% protein, 3.1% fat for a period 4 months.	Paternal obesity and metabolic disorders induced by the WD are inherited by the offspring. F1 males and females: ↑bodyweight, ↓ GT and IS.	[285]^a^
Sprague Dawley (SD) rats	Control (11 kJ/g, 12% fat, 21% protein, 65% carbohydrate) or high-fat diet (HFD; 20 kJ/g, 43% fat, 17% protein, 40% carbohydrate) for 11 weeks	Paternal HFD leads to unique gene signatures in F1 females’ white adipose tissue (RpWAT) and pancreatic islets, mirroring premature aging and chronic degenerative disorders.	[286]^a^
C57BL/6 mice	Control diet (16.1 KJ/g, 14% protein, 21% fat) vs. HFD (Net energy: 19.4 KJ/g, 17% protein, 40% fat) fed for 2.5 months.	F1 males and females: ↓ GT and IS, ↑ adiposity in a sex dependent manner (more in female). F2 females from F1 females born to HFD fed founders: ↑ IR. F2 males from F1 females born to HFD fed founders: ↑ bodyweight, ↓ GT and IS. F2 females from F1 males born to HFD fed founders: ↑ adiposity and IR F2 males from F1 males born to HFD fed founders: no metabolic changes.	[287]^a^
C57BL/6 mice	Control (16.5 kJ/g, 4% carbohydrates, 19% protein, and 17% fat) or HFD (20.7 kJ/g, 32% carbohydrates,19% protein and 49% fat) for 8 weeks	F1 males and females: impaired glucose metabolism and liver steatosis.	[288]^a^
C57BL/6 mice	Control normal protein diet (NPD; 18% casein; 180 g/kg diet; n = 8) versus isocaloric low protein diet (LPD; 9% casein; 90 g/kg diet; n = 8)	In embryos from males fed an LPD, genes in the central metabolic AMPK pathway showed decreased expression. LPD paternal programming increased fetal weight but decreased placental weight, leading to a higher fetal:placental weight ratio. LPD placentas exhibited enhanced expression of transporters for calcium, amino acids, and glucose, as well as elevated levels of epigenetic regulators *Dnmt1* and *Dnmt3L*, affecting maternal and paternal imprinted genes. Fetal skeletal development in response to paternal LPD showed larger skeletons with reduced amount of high mineral density and lower bone apatite maturity.	[15]
Pathogen-free CBA × BALB/c (CBAF1) females and BALB/c males	In natural mating experiment, one to three CBAF1 females (8- to 10-week-old) were paired for mating with intact or seminal vesicle-excised BALB/c males (10- to 20-week-old). Embryos at two-cell or blastocyst stage, collected on gestational day 1.5 or 3.5, were transferred into oviducts or uteri of recipient CBAF1 females, previously mated with VAS or SVX/VAS BALB/c males.	Removing the plasma fraction of seminal fluid on offspring led to impaired conception, late gestational placental hypertrophy, and altered growth and metabolic parameters in offspring, particularly males. Embryos from females mated with males lacking seminal vesicles showed abnormalities, partly alleviated by in vitro culture. The absence of seminal plasma also led to decreased expression of embryotrophic factors (*Lif, Csf2, Il6, and Egf*) and increased expression of the apoptosis-inducing factor *Trail* in the oviduct.	[39]
Boar nutrition	An F0 boar treatment group was fed high methylating micronutrient supplements, while F0 control group received no methyl donors.	Offspring of treated F0 boars were leaner with more shoulder muscle than the control group. The 2 groups showed differential gene expression of Iodotyrosine deiodinase (IYD) gene and DNA methylation.	[63]^b^
Boars in different housing environments	Housed in crates, enriched crates, or pens	Boars housed in enriched crates and pens had more piglets born. The piglets showed fewer skin lesions and exhibited modified responses to skin pressure stimulus.	[62]^b^
Rams on different diet	Evaluating prepubertal methionine feeding in Polypay rams and complex traits, DNA methylation, and transmission of traits to offspring.	Male offspring (F1) from rams that received methionine had reduced body weight and smaller scrotal circumference during puberty.	[64]^b^
Ram nutrition	Pre-pubertal methionine feeding vs control; evaluation of F1, F2 and F3 generations	Sperm from the F1, F2, and F3 generations show altered methylation patterns, accompanied by changes in the scrotal circumference phenotype.	[289]^b^
Bull diet	Supplementing bull diets with Omega 3 (fish oil or flax oil) vs control diet with saturated fatty acids.	The spermatozoa had a carryover effect on the embryos. The blastocyst rate was increased, and blastocyst gene expression was altered.	[65]^b^
Bulls at different age	Semen was obtained from the same bulls at 10, 12, and 16 months of age, and then used for in vitro fertilization (IVF) with oocytes obtained from the same donor cow for each bull.	As the bulls aged, variations in gene expression and methylation were noted, primarily linked to metabolic pathways. Bull age-related sperm-borne miRNAs possibly affected various canonical pathways, including oxidative phosphorylation, mitochondrial dysfunction, and sirtuin signaling pathway, in blastocysts after IVF.	[125]^b^
Bull fertility status	Semen from bulls with high vs. low sire conception rate	Preimplantation embryos originating from high- and low-fertility bulls displayed marked differences in mRNA transcript abundance and methylation in blastocysts.	[290]^b^
Bull seminal plasma	Cows receiving seminal plasma at AI or saline	Exposure to seminal plasma potentially influenced fetal development and affected the birth phenotype. Increased birth weights of calves born from sex sorted semen.	[66]^b^
Bull seminal plasma	Heifers were exposed to seminal plasma from vasectomized bulls before multiple embryo transfers.	At day 14, there was an increase in conceptus length, in addition to modifications of conceptus gene expression.	[67]^b^
Zebrafish gametes and early embryos	Examining DNA methylomes in gametes and early embryo.	Throughout the early stages of embryonic development, the DNA methylation pattern inherited from the father remained stable whereas maternal DNA methylation was stable only up to 16-cell stage. Therefore, zebrafish acquire their DNA methylome via sperm. This indicates a possible pathway for transgenerational epigenetic inheritance through the paternal germline in zebrafish.	[291]
Mature zebrafish	The effects of paternal stress prior to mating on the behavioral and endocrine responses of their offspring.	Stress-related responses in wild-type zebrafish can be influenced by intergenerational disruption through the paternal germ line.	[292]
Roosters from Ross parent stock	The effect of paternal dietary methionine (MET) on offspring carcass characteristics, meat quality, and gene expression.	The MET group had greater eviscerated and thigh muscle yields. Breast muscles in the MET group showed increased lightness and redness, whereas thigh muscles displayed increased redness and yellowness. *BCO1*, *PRKAG2* and *PRDX4* gene expressions were up-regulated in the thigh and breast muscles of tMET group, whereas *PPP1R3A* gene expressions was down-regulated.	[293]

↑ Increased, ↓ Decreased, HFD: high fat diet, GT: glucose tolerance, GTT: glucose tolerance test, IR: insulin resistance, IS: insulin sensitivity. Superscripts ^a, b^ adapted from [1, 12] respectively.

Another recent mouse study reported that the paternal obesity induced by high fat diet (HFD) alters the seminal plasma composition and can impact the immune response of females during conception [31]. It was observed that male mice fed on a HFD had increased seminal vesicle fluid volume and decreased levels of immune-regulatory TGF-β isoforms. Mating with HFD-fed males also led to a weaker post-mating immune response in females, with reduced T-cell activity [31]. These findings highlight the role of paternal obesogenic diet in influencing offspring development through changes in seminal plasma composition. Moreover, all these paternal effects maybe associated with long-term changes in offspring phenotypic traits including future health and disease status. Some of these traits include appetite regulation, energy metabolism, glucose and insulin metabolism, development of muscle, fat, and bone, reproductive efficiency, cardiovascular health, temperament, and social and cognitive skills [1, 41].

A recent study which examined the effects of paternal consumption of a high-protein diet (HPD, containing 43.2% protein) on the endocrine pancreas and the metabolic characteristics of offspring in rats [42] showed that the male offspring born from the HPD-exposed fathers had increased insulin sensitivity but were glucose intolerant. This observation further demonstrates that the paternal nutrition can have a long-lasting impact on the metabolic profile of their progeny in adulthood [33]. Paternal nutrition-mediated programming of offspring lipid metabolism occurs through two distinct routes: sperm which influences offspring genetics and the seminal plasma which can affect the uterine environment, with each pathway affecting offspring lipid metabolism in a distinct manner [43]. The suggested mechanisms underlying this potential impact on offspring health encompass alterations in the seminal fluid composition, sperm DNA methylation, histone composition, small non-coding RNAs (sncRNAs), and sperm DNA damage [12]. The mechanism involving sncRNAs was also previously reported in a study whereby an injection of sncRNAs from sperm of traumatically stressed male mice into fertilized wild-type oocytes was able to replicate the behavioral and metabolic alterations in the resulting offspring [44]. Recently, two independent studies in mice found that cleaved transfer RNAs (tRNAs) in mouse sperm is an inherited epigenetic factor influenced by the male diet that is connected to gene misexpression and disruptions in the offspring’s metabolism [45, 46]. These findings highlight the crucial role of sperm-associated RNAs in mediating the transfer of paternal influence induced even during early life of males to their offspring. Another murine study showed that paternal diets containing fish oil before conception reduced the incidence of necrotizing enterocolitis in the offspring [47]. According to a follow-up study, male mice fed fish oil for one spermatogenic cycle prior to mating showed a decrease in pulmonary inflammation and respiratory pro-inflammatory cytokine expression in the pups [32]. These body of evidence derived from rodent animal models highlight the complex involvement of paternal influences in shaping the offspring health and development.

### Evidence on paternal programming in human studies

In addition to evidence obtained from rodent animal models, there are increasing body of literature from human studies demonstrating the paternal programming of offspring health (Table 2). A study which analyzed umbilical cord blood from 39 newborn children discovered a link between DNA damage and paternal pre-conception smoking rather than maternal passive smoke exposure during pregnancy [48]. Likewise, Haervig and colleagues also reported that sons of smoking fathers, but non-smoking mothers, had a 10% lower semen concentration, and 11% lower sperm count as compared to the sons of non-smoking fathers [49]. They also observed that the children from smoking parents had significantly lower a sperm count as compared to that of non-smoking parents [49]. In another human study, paternal smoking was reported to alter the composition of sperm microRNAs which potentially affects sperm function and embryo development [50]. There are also reports on the negative impact of the paternal obesity in men on offspring health via disrupting hormonal balance, metabolism, and sperm quality, leading to adverse outcomes in the next generation [51, 52]. Specifically, the paternal obesity is associated with insulin resistance, type 2 diabetes, and elevated cortisol levels in umbilical cord blood, elevating the risk of cardiovascular disease in offspring, particularly daughters [53]. Additionally, a metanalysis revealed a link between parental obesity and higher rates of obesity in children [54]. These findings highlight the significant impact of paternal obesity on offspring health in human populations. Another researcher group also examined the effects of paternal dietary supplementation with methyl donors on offspring epigenetic modifications [55]. In agreement with earlier reports, they observed alterations in DNA methylation patterns in the sperm of fathers who received methyl donor supplementation, which correlated with changes in gene expression and metabolic phenotypes in the offspring [56, 57]. These findings together suggest that paternal diet-induced epigenetic changes in the human sperm can influence offspring health and metabolism. Besides, a study conducted by Chen and colleagues investigated how paternal body mass index (BMI) influences offspring growth and whether this relationship is linked to sex-specific paternal programming of cortisol secretion [58]. They employed multivariable regression analyses, considering various maternal confounding factors, and confirmed that there is an association between paternal BMI and male offspring growth. Significant correlations were observed in birth parameters and cortisol levels in male newborns only. These findings suggest that the paternal BMI may serve as one of the risk factors for cardiovascular diseases in male offspring, potentially through sex-specific paternal programming mechanisms. Furthermore, a study conducted in China, analyzing data from the National Free Preconception Health Examination Project involving 529 090 couples, found that paternal alcohol consumption is associated with a higher risk of birth defects, particularly clefts [59]. These findings highlight the importance of reducing paternal alcohol intake before conception to minimize the risk of birth defects in offspring. In another longitudinal study spanning multiple countries, data from 24 168 parents were analyzed to investigate the impact of parental smoking and welding exposures before conception on offspring asthma risk [60]. This study revealed that the offspring from the fathers who smoked before conception had a higher risk of non-allergic early-onset asthma, particularly if the father began smoking before age 15, even if he quit more than 5 years before conception [60]. Additionally, paternal pre-conception welding was independently associated with offspring non-allergic asthma. These findings underscore the significance of pre-conception environmental factors in child respiratory health [61]. Thus, sub-optimal paternal pre-conceptional factors, including poor diets, unhealthy lifestyle choices, and environmental stress, can increase the likelihood of unfavorable outcomes in offspring through both direct (genetic/epigenetic) and indirectly (e.g. maternal uterine environment) pathways.

**Table 2:** Summarized evidence from recent studies illustrating paternal programming effects in humans

Species	Experiment conducted	Links to paternal programming	Citation
Adult offspring (mean age 37 years) and their parents in the Netherlands	A cohort study of adult children (F2) of men and women (F1) born during the 1944–45 Dutch famine.	F2 adult offspring of prenatally exposed F1 fathers had higher body weights (+4.9 kg) and BMIs (+1.6 kg/m2) than those of unexposed F1 fathers.	[294]^a^
Children aged 7–18 years and their parents in China	A cross-sectional survey of the correlation between parental overweight (pre-conceptional BMI) and offspring metabolic syndrome (MS).	Paternal overweight increased the risk of children developing MS by 2.17 times, and was also positively correlated with MS, obesity, and low HDL cholesterol in both boys and girls.	[295]^a^
Newborns and parents in USA	Investigating possible connections between parental obesity before conception (pre-conceptional BMI) and DNA methylation patterns in their offspring.	Elevated paternal BMI was associated with IGF-2 differentially methylated region (DMR) hypomethylation in the umbilical cord blood leukocytes of offspring (β coefficient = −5.28, p = 0.003).	[34]^a^
National Free Preconception Health Examination Project in China, involving 529,090 couples and their pregnancy outcomes and alcohol consumption.	Examined the link between pre-conceptional drinking by fathers and baby abnormalities.	Paternal alcohol consumption was found to be associated with a higher risk of birth defects, especially clefts, after adjusting for confounders.	[59]
Study involving a cohort of 899 father/mother/child triplets in China	Investigated the association between paternal BMI and newborn parameters in 899 triplets, considering sex-specific fetal programming.	The study established a link between paternal BMI and male offspring growth, but not for females. Notably, paternal BMI correlated significantly with several birth characteristics only in male infants, including birth weight, head circumference, and abdominal measures, indicating a gender-specific impact on child growth.	[58]
74 fathers and 51 cord blood samples from Belgium	Examined the impact of paternal consumption of methyl-group donors on the methylation patterns and birth weight of his progeny.	The study found that paternal intake of betaine and methionine was associated with changes in paternal and offspring DNA methylation patterns. Betaine intake positively correlated with paternal global DNA hydroxymethylation and cord blood global DNA methylation. Methionine intake was positively associated with CpG methylation of the *IGF2* DMR in cord blood. Additionally, paternal betaine and methionine intake showed negative correlations with birth weight and birth weight-for-gestational age z-score, while choline intake exhibited a positive association with these birth outcomes.	[55, 57]
A longitudinal study spanning multiple countries, using data from 24,168 parents.	Analyzed the impact of parental smoking and welding exposures before conception on offspring asthma risk	Offspring of fathers who smoked before conception had a higher risk of non-allergic early-onset asthma, especially if the father started smoking before age 15, even if he quit over 5 years before conception. Additionally, paternal pre-conception welding was linked to offspring non-allergic asthma.	[60, 61]

BMI: Body mass index. Superscript ^a^ adapted from [12].

### Evidence on paternal programming in livestock animals

Compared to rodent animal models and human studies, the evidence of the paternal programming in livestock animals is relatively limited. However, there are some studies reporting the association of the management practices with the paternal programming of offspring in swine, sheep, and cattle [1]. Given that in livestock, paternal contributions to developmental programming can have a significant impact on the overall herd due to limited number of sires, or even a single sire, mates with numerous females, there is a growing research interest in studying the paternal programming in livestock sector. A recent study in swine reported that boars housed in enriched crates during pre-breeding spermatogenesis sired more piglets than those housed in unenriched crates [62]. The piglets from boars raised in enriched crates exhibited reduced skin lesions and a diminished response to potentially painful stimuli, suggesting an effect of paternal housing conditions on stress/emotional response areas of the offsprings’ brain. Feeding boars with methyl donors (also known as one-carbon metabolites) resulted in alterations in the gene expression, and DNA methylation in gluteus muscle, liver, and kidney tissues, as well as back fat percentage in their offsprings [63]. Similar observations were reported in sheep. Gross and colleagues observed that feeding sibling ram pairs with a diet containing rumen-protected methionine from weaning to puberty affected post-pubertal sperm methylation in their offspring lamb [64]. These authors also observed that the F1 offspring from methionine-fed rams had lower body weight at puberty and smaller scrotal circumference compared to the offspring born from the rams that were not fed methionine [64]. In cattle, adding omega-3 fatty acids from fish meal or flax meal to the diet of Friesian bulls increased the blastocyst rate and changed embryonic gene expression [65]. Another study revealed the link between bull seminal plasma and increased birth weights in calves born after artificial insemination (AI) with sex-sorted semen [66]. A recent study also reported association of bull seminal plasma with the increased fetal length at day 14 of gestation and modified conceptus gene expression suggesting its potential effects on the conceptus [67]. The observations from the swine, sheep, and cattle studies discussed above highlight specific components or a combination of factors within the semen are likely responsible for eliciting paternal programming responses in livestock animal species.

### Mechanisms involved in transferring the paternal effects to offspring

The evidence derived from rodents, humans, and livestock animal species discussed above demonstrate that paternal factors could have important and long-lasting effects on their offspring health and development. The underlying mechanisms through which paternal effects are transferred into offspring are believed to be associated with sperm and seminal plasma-specific pathways including seminal plasma cytokines, epigenetic signatures, and oxidative stress, which are detailed in the subsequent subsections.

#### Seminal plasma: Compositions and functions

The semen is a complex fluid essential for reproduction in males, and it is primarily consisted of spermatozoa and seminal plasma [68], and it also harbors microbial community [21, 25, 69]. Spermatozoa, produced in the testes, are specialized cells responsible for fertilizing the female egg. The seminal plasma, which makes up the majority of the semen, is a complex biological fluid mainly composed of secretions from various accessory sex glands such as the seminal vesicles, prostate, and bulbourethral gland, as well as components from the seminiferous tubule lumen, epididymis, and vasa deferentia [70]. Among the male accessory glands, seminal vesicles contribute the cytokines, prostaglandins, and fructose, while the prostate glands secrete fluids aiding sperm maturation, semen liquefaction, and motility [71, 72]. Bulbourethral glands produce lubricating substances that facilitate sperm transfer efficiency. Upon ejaculation, sperm cells pass through the ejaculatory ducts where they mix with the glandular secretions to form semen. In addition to biopolymers like cell-free DNA, RNA, microRNAs, peptides, proteins, and oligosaccharides, the seminal plasma contains lipids, glycans, inorganic ions, and tiny metabolites [70, 73]. The seminal plasma thus contains all the essential components that are crucial for the sperm cell function and male fertility. High levels of fructose and lipids in the seminal plasma provide energy for spermatozoa [71], while minerals such as zinc and selenium support spermatogenesis and antioxidant activity [74, 75]. The alkaline nature of the semen, which is mainly maintained by polyamines, supports the sperm cell survival in the acidic vaginal environment [76]. The seminal plasma serves multiple functions in fertilization such as nourishing sperm cells, influencing their functionality, mediating the interaction between the sperm cells and the female reproductive system, particularly with the immune system present in the female reproductive tract. While some studies suggested the limited influence of the seminal plasma components on the fertilization due to their inability to pass through cervical mucus [77, 78], recent research challenges this dogma. Some studies indicate that the seminal plasma contains immunomodulatory factors that can prevent the female immune system from attacking sperm cells, and thereby promote successful fertilization and early embryo development [79–82]. In mice, the removal of the seminal vesicle decreased the proportion of spermatozoa exhibiting progressive motility [83]. Such alteration could significantly impact the fertilization success and lead to a marked reduction in conception rates [83, 84]. Due to its impact on sperm function, motility, transport, and interactions with the female reproductive tract, the seminal plasma can have important role in the fertilization process [85]. Currently, there is growing body of evidence showing sperm genomic (epigenetic) and the seminal plasma work together to actively participate in fertilization and, more importantly, in the transmission of paternal impacts on the health of offspring through the mechanisms including the cytokines, epigenetic modications, and oxidative stress.

#### Cytokines

Cytokines are chemical mediators that regulate homeostasis of immune cell and coordinate signal-dependent immune responses and inflammation [86]. Within cytokine signaling pathways, multiple regulatory checkpoints are incorporated, often involving feedback inhibition. This mechanism facilitates the transition of tissues to a relatively tranquil non-inflammatory and physiological state of immunotolerance [87, 88]. They play crucial functions in controlling reproductive processes such as sperm maturation, fertilization, and embryo implantation, alongside regulating various physiological processes including cell growth, differentiation, and communication within the immune system [89, 90]. Cytokines are produced by a wide range of cells, including immune cells, and exert their effects by binding to specific receptors on target cells [91–93]. Evidence shows that the semen contains cytokines such as transforming growth factor-β (TGF-β), interleukins (ILs), tumor necrosis factor-alpha (TNF-α), and interferons (IFNs) [81, 94, 95]. Seminal plasma cytokines play a critical role in sperm maturation as well as their functionality in the female reproductive tract. For instance, elevated levels of pro-inflammatory cytokines, such as TNF-α are associated with compromised semen quality, sperm function, and DNA integrity [96, 97] potentially compromising fertilization success and embryo development. Moreover, anti-inflammatory cytokines such as TGF-β, interact with the maternal reproductive tract to modulate immune responses and inflammatory processes which prepares uterine tissue for supporting fertilization and embryo implantation [90, 98]. Simultaneously, cytokines enhance the sperm cell viability and improve fertilization, thereby collectively fostering an optimal reproductive outcome. Therefore, seminal cytokine profiles are influenced by factors including the microbial community present in the male reproductive tract and mostly likely by the seminal microbiota. A recent study examined the cytokine secretory inhibitors and microbial biofilms in semen samples from healthy and infertile people [99]. The results demonstrated variable cytokine inhibitor levels whereby *Staphylococcus* increased cytokine inhibitor levels in healthy people, meanwhile *Enterococcus* and *Corynebacterium* reduced cytokine levels in infertile males [99]. Therefore, it is possible that microbial activities influence the seminal cytokines, which may then alter sperm functionality, maternal immunomodulation, and, ultimately fertilization, embryonic development, and could contribute to fetal programming [100].

#### Epigenetic modifications

Although *de novo* mutations in male germlines are often cited for paternal-induced transgenerational phenotypic inheritance, the limited mutation rates indicate the participation of other additional mechanisms [101, 102]. Morover, the genetic mutation hypothesis also insufficiently explains the inheritance of male phenotypes to offspring in genetically identical animals without exposures as reported in mice model [103, 104]. Therefore, it has been suggested that non-genomic mediators, such as epigenetic modifications in mature sperm could influence the male germline upon delivery to the oocyte at fertilization [104, 105]. Epigenetic signatures encompass heritable and stable alterations in gene expression, occurring via changes in the chromosome structure rather than in the DNA sequence [106]. Epigenetic modifications are chemical alterations to DNA bases and changes to the chromosomal superstructure in which DNA is packaged which regulate gene expression and silencing without changing the underlying DNA sequence [107]. In summary, DNA, which carries a negative charge, coils around a positively charged histone protein octamer which has a pair of histone proteins H2A, H2B, H3, and H4 [108]. The nucleoprotein complex, known as a nucleosome, forms the basic unit of chromatin [109]. Nucleosomes along a continuous DNA polymer are joined by linker DNA and complex, which is supported by the histone protein H1. Chromatin aggregation produces chromosomes that reside in either loose, transcriptionally active euchromatin or dense, transcriptionally inactive heterochromatin states [110]. Chemical alterations to histone proteins can cause the creation of either euchromatin, which allows gene expression, or heterochromatin, which inhibits gene expression. DNA methylation, histone modification, and non-coding RNA-associated gene silencing are three separate epigenetic pathways [107]. DNA methylation is catalyzed by DNA methyltransferase enzymes, involves adding a methyl group directly to a cytosine nucleotide within a cytosine-guanine sequence (CpG), often forming CpG islands. CpG islands, primarily found in gene promoter regions, are targets for this epigenetic modification [111]. Methylated cytosines within promoters recruit gene suppressor proteins, reducing interaction with transcription factors, leading to gene silencing [112]. Additionally, cytosine methylation drives heterochromatin formation, preventing transcriptional machinery interaction, further silencing genes [113]. Another layer of epigenetic mechanism involves modifications to histone proteins, including acetylation, methylation, phosphorylation, and ubiquitylation that affect DNA-histone interactions within nucleosomes. Histone acetylation promotes transcription by loosening DNA-histone interactions, while histone methylation, phosphorylation, and ubiquitylation have complex and varying impacts on gene expression [114–116]. The latest proposed epigenetic mechanism involves non-coding RNA (ncRNA)-linked gene silencing. These are functional RNA molecules, including microRNAs (miRNAs), short interfering RNAs (siRNAs), and long non-coding RNAs (lncRNAs), regulate gene expression [117, 118] and potentially contribute to phenotypic diversity [119]. While their exact role is still not fully understood, ncRNAs are implicated in DNA methylation, histone modifications, and gene silencing, potentially through the formation of heterochromatin [120]. These modifications can be influenced by diets, environmental factors and lifestyle choices which make them potential mediators of paternal effects during embryonic development [41]. Sperm experience DNA methylation and histone modifications mainly in the testicle, while miRNA loading likely happens later, potentially in the epididymis [121, 122]. Research indicates that miRNAs found in the testicular parenchyma may also be present in epididymal segments, therefore suggesting the testicle as their potential loading site [123]. Studies on bull sperm show fluctuating miRNA levels as sperm traverse the reproductive tract, with notable increases observed in the epididymis [122, 124, 125]. Therefore, histone modifications, non-coding RNAs and changes in DNA methylation patterns during spermatogenesis likely regulate spermatic gene expression at fertilization and during early embryonic development thereby influencing various physiological processes and offspring phenotypic traits.

#### Oxidative stress

Oxidative stress arises from an imbalance between reactive oxygen species (ROS) production and antioxidant defenses in cells [126, 127]. The sperm, with their high polyunsaturated fatty acid content and limited antioxidant capacity, are particularly susceptible to oxidative damage [128]. Elevated ROS levels can harm sperm DNA, proteins, and lipids, affecting their quality and function [129, 130]. This damage may result in decreased sperm cell motility, impaired DNA integrity, and reduced fertilization potential, impacting embryo development and offspring health. Various factors such as heat stress, obesity, hypertension, insulin resistance, dietary changes, sperm cryopreservation and psychological disorders can trigger oxidative stress in the sperm cell, most likely through mitochondrial and enzyme-mediated mechanisms within the cells [128, 131]. A recent study reported that exposure to ionomycin and hydrogen peroxide triggers oxidative stress, leading to heightened production of reactive oxygen species (ROS), elevated thiol oxidation levels and diminished sperm viability, mitochondrial membrane potential, and motility [132]. These alterations also resulted into a decrease in microtubule-associated protein light chain 3 (LC3-I) levels, indicating the activation of autophagy in response to oxidative stress. Moreover, ionomycin treatment leads to elevated levels of LC3-II, autophagy-related 16 proteins, and phosphorylated form of AMP-activated protein kinase. Moreover, blocking autophagy in sperm exposed to oxidative stress results in compromised sperm quality and metabolic parameters, accompanied by increased cell death markers [132]. Consequently, sperm DNA damage caused by oxidative stress may contribute to paternal programming effects on offspring, affecting their health and development [133–135].

In summary, epigenetic modifications in the sperm, oxidative stress, and seminal cytokines are relatively known mechanisms mediating the paternal programming of offspring health. However, the emerging role of the seminal microbiome as a potential contributor in the mechanisms transferring paternal influences to offspring still necessitates further investigations into its role in reproductive biology and beyond.

## The seminal microbiome and paternal programming

### Seminal microbiota

Currently, there is a growing interests in the seminal microbiome research due to its potential implications not just for male reproductive health but also for female reproductive health and fertility [25, 136]. Recently, the seminal microbiome has been proposed to be involved in transmission of paternal programming effects and hence has emerged as a novel contributor to the DOHaD [1]. Interestingly, mammalian semen is a complex biological fluid that supports sperm function as well as provides a conducive environment for the growth and establishment of micro-organisms [137]. Following the recent advent of high-throughput sequencing techniques, the conventional notion that the male reproductive tract should be entirely devoid of microbial cells and that microbes present in the semen are inherently pathogenic agents, has encountered a paradigm shift [22, 24]. This transformation stemmed from the recent identification of a diverse and relatively complex microbial community in the semen of healthy males using both culture-independent high-throughput sequencing and culture-dependent methods [138, 139] (Tables 3 and 4).

**Table 3:** Common seminal microbiota identified in different animal species using both high-throughput sequencing-based and culture-based methods reported by recent studies.

Species and	Sampling and processing techniques	Genera reported	Key findings	References
45 beef bulls (1–6 years old) in United States	Semen with satisfactory and unsatisfactory spermiograms were collected by electroejaculation in a sterile artificial vagina. Processed for 16S rRNA gene sequencing (V4); Illumina MiSeq	*Escherichia-Shigella, Bacteroides, Corynebacterium 1, Streptococcus, and Histophilus, Gemella* were found in samples with satisfactory spermiograms. *S5-A14a, Bacteroides, Escherishia-Shigella, Gemella, Enterococcus, and Histophilus* were reported in semen samples with unsatisfactory spermiograms.	*Bacteroides, S5-A14a, Trueperella, Methanosphaera, and Methanobrevibacter* were more abundant in bulls with excellent spermiograms. Meanwhile *Veillonellaceae, Campylobacter, and Methanobacterium* were found in greater numbers in bulls with poor spermiograms. There were notable microbial interactions in their sample set for both excellent and unsatisfactory semen samples.	[140]^a^
18 Holstein bulls of known fertility (3–10 years old) in Sweden	Semen correlated with fertility, either positively or negatively, were collected by electroejaculation using a sterile artificial vagina. Processed for 16S rRNA gene sequencing (V4); Illumina MiSeq	*Porphyromonas, Fusobacterium, Ruminococcaceae UCG-010, Fastidiosipila, Ruminococcaceae UCG-005, Cutibacterium, Histophilus, Oceanivirga, Corynebacterium 1, Campylobacter, W5053, Dyella, Staphylococcus, Lawsonella, Helcococcus, Bacteroides, Capnocytophaga, Curvibacter, Kingella,* and *Enhydrobacter*	*Curvibacter, Rikenellaceae, RC9-gut-group, Cutibacterium, Ruminococcaceae UCG-005, Ruminococcaceae UCG-010, Staphylococcus, and Dyella spp.* were shown to be negatively correlated with fertility. Among bulls with low fertility, the genera *W5053* and *Lawsonella* were more abundant.	[142]^a^
55 Holstein Friesian bulls in Slovakia	Semen samples collected using a sterilized artificial vagina. Samples processed for 16S rRNA gene sequencing (V4); Illumina MiSeq	*Fusobacterium, Actinobacillus, Bacteroides, Cutibacterium, Staphylococcus,* and *Prevotella*	The microbiome makeup could be divided into two separate clusters: Cluster 1 had an elevated proportion of Actinobacteria and Firmicutes, whereas Cluster 2 had a high predominance of Fusobacteria.	[141]^a^
38 crossbred bulls(yearlings) in the United States	Using electroejaculation, semen samples were collected prior to breeding and after a 28-day breeding period. 16S rRNA gene sequencing (V3-V4); Illumina NovaSeq 6000	Semen collected before breeding: *Fusobacterium, Prophyromonas, Oceanivirga, and Corynebacterium* Semen collected after 28 days of mating: *Fusobacterium, Prophyromonas, Oceanivirga,* and *Corynebacterium*	After the 28-day breeding phase, the semen samples showed an increase in microbial richness and diversity. Seminal microbiota was not affected by a moderate vs. a high rate of gain dietary treatment.	[24]^a^
Five equine stallions (7–17 years old) from Sweden	Semen samples collected using a sterile artificial vagina with a reusable liner and filter and sterile collecting flask. The samples processed for analysis by Bruker Biotyper MALDI-TOF	*Staphylococcus, Bacillus, Corynebacterium, Micrococcus, Fusobacterium, Mycoplasma, Pantoea, Brevibacillus, Paenibacillus, Actinetobacter*, and *Aerococcus*	The skin and mucous membranes are the source of 50% of the bacterial taxa identified. membranes and the skin All samples were found to have *Staphylococcus sp.* All extended samples and the extender had *Micrococcus spp.* The frequency of bacterial identification varied across the subjects, and it was unclear how the identified bacteria affected the quality of the sperm.	[143]^a^
16 boars in the United States	Semen samples were characterized using 16S rRNA sequencing	*Clostridium, Alkaliphilus, Corynebacterium, Ruminococcus, Mannheimia, Psychrobacter, Moraxella,* and *Brenneria.*	There were negative relationships between sperm motility and the relative abundance of *Prevotella, Ruminococcus,* and *Bacteroides.*	[145]
Six Murciano-Granadina goat bucks in Spain	Samples collected into artificial vagina both during the breeding and non-breeding seasons. Samples processed for 16S rRNA gene sequencing (V3-V4); Illumina MiSeq	During the breeding season: *Ureaplasma, Oceanivirga, Mannheimia, Fastidiosipila, and Sphingomonas* During the non-breeding season: *Ureaplasma, Lactobacillus, Bradyrhizobium, Chloroplast, Clostridium_sensu_stricto_1, and Romboutsia*	During both the breeding and non-breeding seasons, Uraplasma was the most prevalent genus. Semen microbiota vary with the seasons, however it remained consistent for 7 days within a given season. *Faecalibacterium* and *Spingomonas* are potential indicators for the semen quality in goat bucks.	[146]^a^
9 poodle dogs from the same owner in South Korea	The effect of oral administration of three commensal *Lactobacillus species* on the qualitative sperm parameters of healthy normozoospermic dogs. Samples analyzed using Illumina MiSeq platform 16S (V3-V4).		The relative abundance of *Fusobacterium perfoetens* and *Anaerobiospirillum succiniciproducens* reduced, while *Limosilactobacillus reuteri* increased. Oral supplementation of commensal *Lactobacilli* improved sperm parameters such as total and progressive motility, acrosome integrity, and other kinematic parameters.	[148]
9 samples from captive collared peccaries (*pecari tajacu*)	Foreskin mucosa and ejaculate were collected from adults. The samples prepared for culture.	*Corynebacterium* sp*.* *Staphylococcus* sp*.* *Dermabacter* sp *Arcanobacterium* sp*.*, and *Bacillus* sp*.*	Increase in abundance of *Corynebacterium* sp. within collared peccary semen had an adverse impact (*P* < 0.05) on sperm membrane integrity.	[147]
60 British United Turkeys (BUT breed).	Investigated the presence of commensal bacteria in the reproductive fluid and their influence on semen. Analyzed using MALDI-TOF.	*Escherichia coli, Proteus mirabilis, Staphylococcus lentus,* and *Citrobacter braakii*	The presence of bacteria primarily affected sperm motility, membrane integrity, and mitochondrial function. This damage was strongly linked to oxidative stress and inflammation.	[149]

^a^Adapted from [25].

**Table 4:** Common seminal microbiota identified in human semen using high-throughput sequencing-based methods.

Species and	Sampling and processing techniques	Genera reported	Key findings	References
19 sperm donors and 58 patients seeking infertility treatments in China.	Seminal fluids were obtained from 77 individuals ranging in age from 18 to 40. Semen samples were analyzed by pyrosequencing the V1–V2 region of the 16S rRNA genes from their total genomic DNA.	The most prevalent bacteria in the semen were: *Lactobacillus, Corynebacterium, Streptococcus, Staphylococcus, Prevotella, Finegoldia, Anaerococcus, Peptoniphilus, incertae sedis XI (family), Veillonella, Pelomonas, Porphyromonas, Acidovorax, Atopobium, Ureaplasma, Bradyrhizobium, Aerococcus,* and *Gemella, Granulicatella*	Various forms of bacteria were identified in semen, but no significant differences were revealed between sperm donors and infertility. A significant negative correlation between sperm quality and *Anaerococcus* presence.	[22]
93 human patients from infertile couples in Switzerland.	Ejaculate from Swiss males attending a reproductive clinic – 25 with normal spermiograms and 68 with at least one aberrant spermiogram parameter. Samples processed for 16S rRNA gene sequencing	*Corynebacterium, Prevotella, Lactobacillus, Streptococcus, Staphylococcus, Planococcaceae, Finegoldia, Haemophilus, Burkholderia*	*Prevotella* relative abundance increased in samples with impaired sperm motility, while Staphylococcus was high in the control group. Lactobacillus has shown protective effects on semen parameters.	[21]
96 healthy Taiwanese men apart from inability to conceive for >1 year	36 normal semen samples and 33 semen samples with abnormal clinical values. Semen collected into a sterile bottle after 3–5 days of abstinence. Samples processed for 16S rRNA gene sequencing	*Lactobacillus, Pseudomonas, Prevotella, Gardnerella.*	High dominance of *Lactobacillus* correlates positively with improved semen quality. *Lactobacillus* may be a useful probiotic for preserving semen quality and for reducing the detrimental effects of *Prevotella* and *Pseudomonas.*	[138]
42 semen samples from idiopathic infertile Spanish patients and 14 control seminal microbiota were analyzed.	The MinION platform used to perform full-length 16S rRNA gene sequencing and compared with those analysed using the MiSeq platform	Most abundant genera were *Peptoniphilus, Finegold-ia, Staphylococcus, Anaerococcus, Campylobacter, Prevotella, Streptococcus, Lactobacillus,* and *Ezakiella.*	Prominent bacterial genera revealed by the ONT MinION platform were found to be consistent with Illumina MiSeq results for the same cohort. However, a few bacterial genera, such as Staphylococcus, *Lactobacillus, Corynebacterium, Peptostreptococcaceae,* and *Moraxellaceae*, showed variations in abundance across the two sequencing platforms.	[69]

The 16S rRNA gene amplicon-sequencing based studies reported that the bovine semen harbors a relatively diverse and complex microbial community, with the most prevalent bacterial phyla being *Firmicutes, Proteobacteria, Fusobacteria, Actinobacteria,* and *Bacteroides* [24, 140–142]. Our research group identified over 6900 distinct amplicon sequence variations (ASVs-proxy to species) and 28 different bacterial phyla in the semen samples of yearling beef bulls collected at three developmental stages, with a total bacterial concentration ranging between 8.8 and 9.0 log_10_ 16S rRNA gene (V4) copies per milliliter of semen. We also cultured, isolated, and identified over 350 bacterial isolates from 49 bacterial genera using multiple culture media under both aerobic and anaerobic conditions [24]. Interestingly, several bacterial species identified in the bovine semen are known pathogens involved in bovine diseases such as liver abscess (*Fusobacterium necrophorum* and *Trueperella pyogenes*), bovine respiratory disease (*Histophilus somni* and *Mannheimia varigena*), foot rot (*Fusobacterium gastrosuis*), mastitis (*Arthrobacter gandavensis*), and abortion (*Trueperella abortisuis* and *Bacillus cereus*) [24]. This suggests that the microbiota in the bovine semen is relatively complex and diverse which may evolve over the duration of mating seasons and play certain crucial roles in the process of reproduction.

A study conducted in equine stallions, utilizing both Matrix-assisted laser desorption ionization time of flight mass spectrometry (MALDI-TOF) and 16S rRNA sequencing also revealed a diverse microbial community in the horse semen, including *Actinobacter, Micrococcus, Fusobacterium, Mycoplasma, Pantoea, Brevibacillus, Paenibacillus,* and *Aerococcus* [143, 144]. A recent study employing 16S rRNA sequencing identified 6357 operational taxonomic units (OTUs) across 89 samples of freshly extended boar semen, indicating the presence of rich bacterial populations in the boar semen [145]. Moreover, it was also noted that the relative abundance of *Prevotella*, *Ruminococcus*, and *Bacteroides* had significant negative associations with the sperm motility. Research in Murciano-Granadina goat bucks utilizing 16S rRNA gene sequencing reported a relatively diverse and intricate seminal microbiota with distinct community structures between breeding and non-breeding seasons. During the breeding season, predominant genera included *Ureaplasma*, *Oceanivirga, Mannheimia*, *Fastidiosipila,* and *Sphingomonas*, whereas the non-breeding season showed *Ureaplasma*, *Lactobacillus, Bradyrhizobium*, *Chloroplast*, *Clostridium sensu stricto* 1, and *Romboutsia* as the most abundant species [146]. The presence of a relatively rich and diverse seminal microbiota has also been documented in many other animals, from wild animal species like collared peccaries (*pecari tajacu*) [147] to domestic dogs [148], to poultry [149], and extending to humans (see full list in Tables 2–4).

In humans, the seminal microbial community is comprised mostly of bacteria (71.3%), with *Bacillus, Staphylococcus, Mycobacterium*, and *Streptococcus* being the most abundant genera (Table 4). Eukaryotes (27.6%) and viruses (1.1%) are also reported to be present in the seminal microbial community of human semen [150]. In a recent study involving 96 patients seeking fertility services, characterization of the semen using the 16S rRNA gene sequencing identified *Lactobacillus, Pseudomonas, Prevotella,* and *Gardnerella* as dominant bacterial genera. In addition, a positive correlation was observed between elevated *Lactobacillus* dominance and enhanced semen quality, implying that *Lactobacillus* could serve as a potential probiotic for preserving semen quality and counteracting the effects of *Prevotella* and *Pseudomonas.* A recent human investigation by Baud and colleagues similarly found that *Lactobacillus* has protective benefits on sperm parameters, whereas an increased relative abundance of *Prevotella* was observed in samples with lower sperm motility [21]. These findings highlight the importance of understanding the potential roles that the seminal microbiota may have in maintaining reproductive health and its potential implications for fertility and beyond, in both animals and humans.

Most of the available data from human and animal models is limited to taxonomic characterization of the seminal microbiota [24, 25, 69, 151]. However, the origin of the seminal microbiota remains to be elucidated. The potential origins of the seminal microbiota could be from multiple sources including tissues responsible for producing, transporting, or delivering seminal components (e.g. the seminiferous tubules, the epididymis), the circulating blood supply, and the transfer of vaginal and uterine bacteria to the male reproductive tract during copulation [152]. Some anatomical sites such as prostate glands, seminal vesicles, ductus deferens, testis, bulbourethral glands, the glans penis, and the penile shaft might be involved in seeding semen with microbes [25, 153, 154]. In addition, accessory structures like the seminal vesicles, being a vital component of the male reproductive tract, likely contribute to the seminal fluid composition whose functions go beyond just delivering sperm to fertilize oocytes [73, 79, 155]. The seminal fluid contains an array of bioactive molecules, signaling factors, microbiota and microRNAs that are transferred to the female reproductive tract to facilitate conception and pregnancy [79]. These seminal components are believed to initiate controlled inflammation and alteration of transcription in female reproductive tract tissues, which could ultimately influence embryo development and trigger immune adaptations to enhance receptivity, perhaps ultimately enhancing offspring health [79].

Besides microbial seeding sources, the composition of the seminal microbiota is potentially affected by several factors such as paternal diet, host age, sexual activity, and reproductive hormone status. Male mice fed on a HFD have been reported to have an altered seminal microbiota. The HFD mice exhibited increased relative abundance of *Corynebacterium* spp. and decreased *Acinetobacter johnsonii*, *Propionibacterium acnes,* and *Ammoniphilus* and *Bacillus* spp. in their semen as compared to that of control diet fed mice [156]. Moreover, recent research indicates a similarity in bacterial species between the microbial communities associated with testis and gut, and both of these communities are adversely affected by an HFD. In addition, the seminal microbiota could potentially be influenced by management factors such as housing environment [157, 158], temperature [153], antibiotic exposure [159, 160], and vaccination status [161]. In summary, recent research highlights the diverse microbial community present in semen, challenging the traditional view of the male reproductive tract as sterile. The structure and diversity of the seminal microbiome are potentially influenced by several factors like age, diets, and several lifestyle choices. These factors may have an impact on the quality of semen and fertility outcomes, which can have consequences for the reproductive health of both male and female in addition to resulting offsprings. Therefore, understanding the role of the seminal microbiome offers insights into fertility preservation in humans and management strategies for both animals in addition to possible transgenerational impacts of seminal microbiome.

### Potential role of the seminal microbiome in male reproductive health and fertility

The overarching goals of the seminal microbiome research vary depending on the mammalian species being investigated. In humans, the emphasis is on preventing sexually transmitted illnesses and their effects on sperm quality and fertility. However, in livestock, the goal has been to enhance sperm quality for AI as well as minimize pathogen transfer to females and their offspring [150]. Given the intimate connection between the seminal microbiome and sperm quality, it is highly likely that the seminal microbiota is also involved in determining male reproductive health and fertility. In the past decade, several studies have reported correlations between bacterial species found in human semen and the sperm functions and the male fertility [162–165]. Some examples of common seminal microbiota identified in different animal species and humans using both high-throughput sequencing-based methods in recent studies is presented in Tables 3 and 4 resepctively. For instance, Bacterial species such as *Enterococcus faecalis* and some species within *Corynebacteria, Prevotella* and *Anaerococcus* genera have been reported to have inverse correlation with sperm motility [166]. A greater prevalence of *Pseudomonas* was reported in individuals with a sperm disorder known as oligoasthenospermia [166]. Likewise, higher abundance of *Ureaplasma urealyticum* was found in the semen of infertile males [162]. The presence of *Moraxella, Brevundimonas,* and *Flavobacterium* was also negatively correlated with sperm DNA fragmentation [167]. Furthermore, a recent study reported that patients with oligoasthenoteratozoospermia and/or seminal hyperviscosity had a two-fold increase in the prevalence of *Pseudomonas, Klebsiella, Aerococcus, Actinobaculum,* and *Neisseria* spp. [168]. Given that sperm cells make up only 2–5% of an ejaculate, it is likely that the microbiota plays a role in determining the seminal plasma viscosity, which may influence the male fertility [169]. Another human study showed that azoospermic patients with the reduced abundance of several seminal bacterial taxa compared with healthy control men had increased expression of genes related to glycan biosynthesis, nucleotide metabolism but downregulated expression of genes linked to lipid and amino acid metabolism [170]. Similarly, the semen of male donors who were normozoospermic had distinct beta diversity from donors who had asthenozoospermia and oligoasthenospermia [171]. Thus, the evidence discussed above support the notion that the presence of certain microbes in the male reproductive tract, including semen, can have significant negative impacts on sperm quality, male fertility as well as risk of metabolic and immune diseases in male.

While the negative effects of pathogenic bacteria on sperm parameters such as sperm count, motility, morphology, and DNA integrity are relatively well established, there is increasing appreciation of the possible benefits of the seminal commensal microbes in male infertility treatment. In a study comparing the seminal microbiota of normal and leukocytospermic human males in China, the presence of *Lactobacillus* in semen was linked to better sperm quality and improved male fertility [172, 173]. It was also reported that semen samples colonized by *Lactobacillus jensenii* had significantly improved *in vitro* fertilization (IVF) success rates [174]. Furthermore, in a human study involving 97 assisted reproductive technology (ART) couples and 12 healthy (non-ART) couples, it was noted that partners with *Lactobacillus crispatus-*dominated vaginal microbiota and *Acinetobacter-*dominant seminal microbiota showed a greater ART success rate [175]. A normal semen parameters in humans were linked to a higher prevalence of *Staphylococcus* [21]. Another human study on the seminal microbiota and the *in vitro* fertilization environment observed that certain semen-borne bacteria can affect embryo quality. For example, the abundance of *Alphaproteobacteria* and *Gamma proteobacteria* were associated with lower-quality embryos, whereas *Enterobacteriaceae* were associated with to higher-quality embryos [176]. In horses, the relative abundance of seminal *Peptoniphilaceae* was reported to be positively correlated with total sperm motility [26]. Therefore, these evidence imply that maintaining a balanced microbiota in the male genital system is critical for establishing good reproductive health [177]. It has been suggested that imbalances in the community structure and function of the microbial community in the male reproductive tract, including the semen, may contribute to a variety of reproductive problems such as infertility [152], orchitis, epididymitis or epididymo-orchitis, prostatitis, and urethritis reviewed elsewhere [178, 179] Given the presence of a diverse and abundant microbial population in semen which is influenced by several factors, it is highly possible that the contribution of the microbiota goes beyond just influencing reproductive health and fertility.

### Interaction of the seminal microbiome with the vagino-uterine microbiome

Copulation is an essential aspect of vertebrate reproduction which also serves as an avenue for the exchange and interaction between the microbial communities associated with male and female reproductive systems [137]. Several studies have proposed semen as a potential medium for the transfer of microbes from the male reproductive system to the female reproductive tract [136, 180, 181]. There is also general consensus on the concept of “complementary semino-vaginal microbiota,” which explains the potential transfer of micro-organisms from the male to the female reproductive tract via semen during copulation [25, 136, 180]. A recent study done in infertile couples reported the existence of mutual transfer of bacteria between the male and female urogenital tracts [182]. It was also reported that the penile microbiota closely mirrors the vaginal microbiota of the partner, suggesting the bacterial transmission from female to male, primarily *Lactobacillus* spp. In addition, the seminal microbiome was closely matched with the vaginal microbiota of the partner, with *Prevotella* being the most abundant genera in both samples. In another study, Okwelogu and colleagues identified a total of 282 genera as a common genera between the microbiota of the semen and the vagina of couples. The most predominant genus was *Lactobacillus* (43.86%), followed by *Gardnerella, Veillonella, Corynebacterium, Escherichia, Haemophilus,* and *Prevotella* [174].

**Figure 1:**
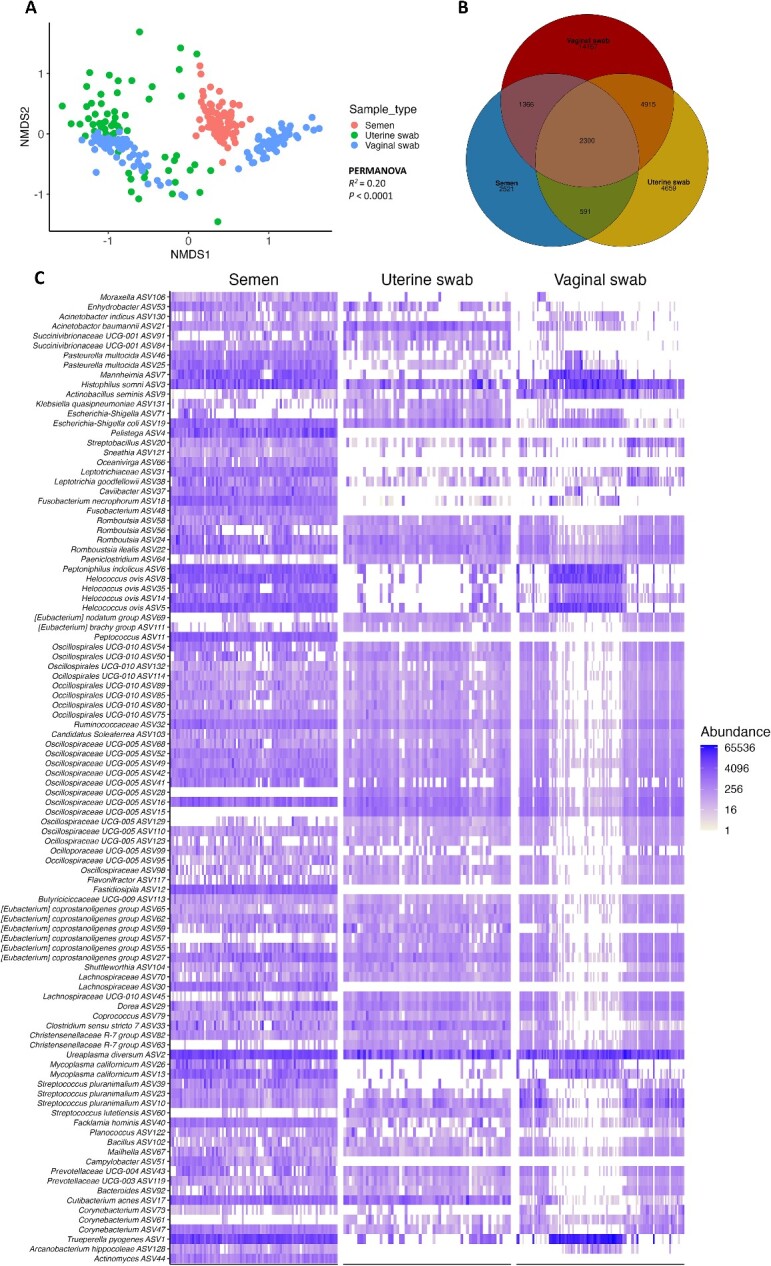
(A) NMDS plot of the Bray–Curtis dissimilarities. (B) Venn diagram showing the number of shared and unique ASVs among the seminal, vaginal, and uterine microbiota. (C) Heatmap showing the 100 most abundant ASVs (log_4_ transformed) overall in the seminal (*n* = 84 samples/yearling bulls), vaginal (*n* = 114/ heifers and cows) and uterine (*n* = 60/cows) microbiota. (Amat et al., unpublished data).

Similar observations have been reported in cattle. Our research group recently examined the similarity between the seminal microbiota of yearling beef bulls [24], the vaginal and uterine microbiota of female beef cattle (heifers and cows) [183]. We found that, despite the community structures were significantly distinct among semen, vagina, and uterus (*P* < 0.0001; Figure 1A), 5.5% of the total ASVs (41,991) were shared between these three microbial communities (Figure 1B); 8.7% of the total seminal ASVs (6878) were shared with the uterine microbiota and 20% were shared with the vaginal microbiota. Furthermore, the heatmap of the 100 most prevalent ASVs (Figure 1C) (Amat et al., unpublished data) showed that the majority of ASVs are present in all three sample types, albeit with considerable interindividual differences in abundances. Several ASVs (ASV3, 19, 24, 32, 16, and 2) were highly abundant in most samples, suggesting the potential existence of “core taxa” shared by the vagino-uterine and seminal microbial communities. It is interesting that potential pathogens like *Ureaplasma diversum, F. necrophorum, H. somni, Mannheimia haemolytica*.*,* and *T. pyogenes* were observed among these potential core taxa. The evidence derived from the human and bovine studies together indicate the presence of shared bacterial taxa between the seminal and female urogenital microbial communities [25, 175, 180, 184–186]. These observations highlight a noteworthy convergence between the seminal and the vagino-uterine microbiome in cattle, characterized by shared bacterial taxa, thereby implying interactions between these microbial communities during the reproductive process.

When the seminal microbiota is transferred to the female reproductive tract, it potentially interacts with the vaginal and uterine microbiota [25]. Recently, it has been postulated that during the post-coital and pre-implantation stages, the seminal bacteria could hitchhike into the female reproductive tract and could form a “transient, combined male and female microbiota [187]. In the female reproductive tract, the immune cells are equipped with pattern recognition receptors that can recognize and detect the microbes including those of the semen origin [188–190]. The microbial recognition by the immune cells then triggers immunological responses and cytokine production [191, 192], which could ultimately influence fertilization and potential pregnancy outcomes [136, 187, 193]. The interaction between the seminal and vagino-uterine microbiota has been proposed to influence conception and embryonic development by modulating the maternal immune system, nutrient or hormonal signaling mechanisms which eventually introduce epigenetic modifications to the vagino-uterine and embryonic cells [25, 28].

Regulatory T cells (Tregs), a vital subset of T lymphocytes, are renowned for their role in immunoregulation and maintaining immune tolerance both in physiological and pathological conditions [194]. Their suppressive function plays a critical role in establishing and maintaining maternal tolerance to fetal alloantigen, essential for ensuring successful pregnancy [195]. Elevated levels of pregnancy-associated hormones, including estrogen, progesterone, and human chorionic gonadotropin, facilitate the recruitment and expansion of Tregs, highlighting their central role in modulating fetal-maternal immune tolerance [196, 197]. A recent human study reported that in a stable relationship, the more the female is exposed to her partner’s semen, the more Tregs are developed to reduce the mother’s anti-fetal immune response [198]. Interestingly, the direction and strength of the immune response from the cervicovaginal epithelial cells are dependent on the maternal genetics and can be influenced by the seminal immune-modulatory prostaglandins and cytokines [199]. Thus, it is reasonable to speculate that *in utero* exposure to microbes, possibly originating from the semen, has been reported to trigger endotoxin tolerance and fetal colonization [198], thereby enhancing the offspring’s resistance to further microbial exposure. Additionally, hormonal fluctuations may be a critical factor influencing the outcome of this microbial interaction. For instance, during pregnancy, hormonal changes are associated with a microbial shift towards a community dominated by *Lactobacillus* species [200, 201]. There is well established complex interaction between the vaginal microbiota and host immune response, along with its metabolic environment [202–204]. For example, lactic acid producers such as *Lactobacillus species* produce both D- and L-lactic acid, but also vaginal epithelial cells also release lactic acid, primarily L-lactic acid, which can cause inflammatory reactions [205]. Elevated D-lactic acid levels in *L. crispatus*-dominated microbiota may affect cervical integrity [206]. All together, these observations suggest that the seminal microbiota can interact with microbial communities within the female reproductive tract and modulate maternal immune responses. Such interaction has the potential for transmitting paternal programming effects to the offspring directly or indirectly via regulating the maternal immune system, oocyte RNAs, or modifying zygotic gene expression, as well as influencing the embryonic transcription program during pregnancy.

### Potential involvement of the seminal microbiome in paternal programming

Over the last two decades, many studies utilizing both rodent and livestock animal models, have provided convincing evidence that the environment encountered by the male during spermatogenesis can significantly affect the phenotype of their offspring [1, 14, 43, 207]. Much as paternal programming provides a compelling view concerning the transgenerational contribution of the multitudes of paternal environmental factors on the DOHaD, the role of the seminal microbiome in paternal programming is yet to be uncovered. Despite the increased appreciation of the existence of the seminal microbiome in both humans and animals [25, 162], its contribution in shaping the developmental and health trajectory of offspring has not been well examined. Recently, the seminal microbiome has been suggested to be either directly or indirectly involved in the potential mechanisms through which paternal effects are transferred to the offspring, along with the better known effects of epigenetic modifications, oxidative stresses, and cytokine-mediated mechanisms [1]. The health conditions of the males during spermatogenesis, such as obesity, nutritional status, age, and lifestyle, could not only influence sperm quality but also influence the seminal microbiota. There are reports regarding the potential influence of chronic stress and mental health conditions such as anxiety, depression and irritable bowel syndrome on the gut-brain axis and the gut microbiota composition that may indirectly affect sperm quality and the microbial community of the semen [208]. For instance, studies in humans demonstrated that dietary protein could influence the composition of the gut microbiota by promoting protein fermentation and absorption [209] and this may extend to the seminal microbiota. The gut microbiota also produce metabolites such as short chain fatty acids (SCFAs), indole derivatives, neurotransmitters and vitamins which potentially have either beneficial or detrimental impacts on the host’s physiological and general health [210]. Moreover, the seminal and fecal microbiota of yearling bulls have been reported to share 6.1% of total ASVs observed in fecal and semen samples. The shared taxa include *Bifidobacterium pseudolongum, Romboutsia ilealis, Clostridium sensu stricto 1*, and *Corynebacterium marinum* [24]. Thus, it is plausible that any alterations in the gut microbiome may influence the seminal microbiota [152, 211]. This subject is reviewed elsewhere [163]. Consequently, it is possible that the seminal microbiota either directly affects the developing spermatocytes by binding to sperm cells or indirectly by metabolites which signal secretory pathways and modulate general host physiology.

A recent study in mice assessed the consequences of a paternal low-protein diet (LPD) on the semen quality, maternal uterine physiology, and the health of offspring [14]. The authors found that sperm from male mice fed the LPD compared to males fed a normal protein diet (NPD) exhibited global hypomethylation, which was linked to decreased expression of DNA methylation and folate cycle-regulatory genes in the testes [14]. In addition, they found that females mated with LPD males displayed attenuated uterine immunological responses, altered cell signaling, and impaired vascular remodeling during pre-implantation compared to the NPD group [14]. Moreover, it was also reported that embryos from LPD-fed males exhibited downregulation of genes involved in AMP-activated protein kinase (AMPK) signaling pathway, which is important in cellular energy homeostasis during preimplantation. The placentas from LPD-fed males also had increased transporter and expression of *Dnmt1 and Dnmt3L* epigenetic regulators [15]. This was further supported by another mice study where the paternal LPD led to alterations in the expression profiles of key epigenetic regulators responsible for DNA methylation, histone modifications, and RNA methylation in the testes of adult F1 males [207]. Given that the seminal microbiota is also likely be affected by the LPD, the seminal microbes could influence availability of substrates for methylation such as methyl or acyl donors [212], it is plausible that the seminal microbiota could participate in semen associated epigenetic modifications. Other research has explored the impact of a HFD on the gut microbiota and showed that a HF and high-sugar diet consistently induced modifications in the gut microbiota, regardless of differences in the host genotype [213–215]. A recent investigation of the gut-testis axis reported that altering the gut microbiota using alginate oligosaccharide increased sperm quality in type 2 diabetic mice, indicating a potential therapeutic approach for T2D-related male subfertility [216]. Given the potential link between the gut and seminal microbiota, such dietary influences could play a role in the transmission of paternal programming effects to offspring through microbiota-mediated epigenetic modification and regulation of embryonic transcriptional program. Recently, Shock and colleagues proposed a complex interplay between diet, gut microbiota, and host epigenetics in influencing host health [217]. They reported that the structure of the gut microbiota determines the availability of substrates for epigenetic modifications such as DNA methylation, histone methylation, and acetylation [217]. These findings have been supported by a recent study which indicated that microbial metabolites like butyrate can impede crucial epigenetic enzymes, including histone deacetylases [218]. Therefore, it is possible that dietary factors can affect the seminal microbiota and their metabolites, which potentially serve as a route for transfer of paternal programming effects to offspring via epigenetic modifications.

The seminal microbiota could also influence the sperm motility directly through attachment to spermatozoa as well as by initiating release of inflammatory cytokines or ROS that affect sperm function or cause apoptosis [136, 139]. Following breeding, the introduction of the semen and the seminal microbiota, along with cytokines including interleukin-6 (IL-6), tumor necrosis factor-alpha (TNF-alpha), and interleukin-1beta (IL-1beta), into the uterus triggers the recruitment of immune cells such as the migration of polymorphonuclear leukocytes to the uterine lining in response to the presence of cytokines within the female reproductive tract [219] reviewed by [220]. Activated polymorphonuclear leukocytes (PMNs) release DNA and histones, generating neutrophil extracellular traps that eliminate microbes and spermatozoa, allowing the endometrium to be restored for embryo reception [221]. Therefore, it is highly likely that the influence of the seminal microbiota has a long-term impact on pregnancy outcomes and the health of the offspring.

A recent findings from our studies suggest that similar microbiota including some pathogenic species exist in the seminal microbiota of healthy bulls, the vagino-uterine microbiome of healthy cows, and the different body sites of the newborn calves [24, 25, 48, 222]. It has also been reported that alteration in the diversity of the seminal microbiota and modifications of related metabolic pathways, such as those pertaining to the metabolism of lipids, glucose, and cholesterol [15], may affect a male’s general health and reproductive performance. These changes can also have an impact on the health of the mother, the offspring, and future generations [223]. Therefore, seminal microbial dysbiosis, which can be a common sequala of antibiotic treatment, could impact on paternal programming of offspring health outcomes. Furthermore, a study on the effect of seminal microbiota and the *in vitro* fertilization environment in humans noted that certain semen-associated bacteria affect embryo quality [224]. Moreover, presence of *Alphaproteobacteria* and *Gamma proteobacteria* were recently shown to be linked to lower-quality embryos, but *Enterobacteriaceae* were linked to higher-quality embryos [176]. These findings suggest that the seminal microbiota may not only be involved in altering sperm quality but also have impacts on embryo quality and ultimately contribute to the transfer of paternal programming to the offspring.

## Possible mechanisms through which the seminal microbiome mediates paternal programming

**Figure 2:**
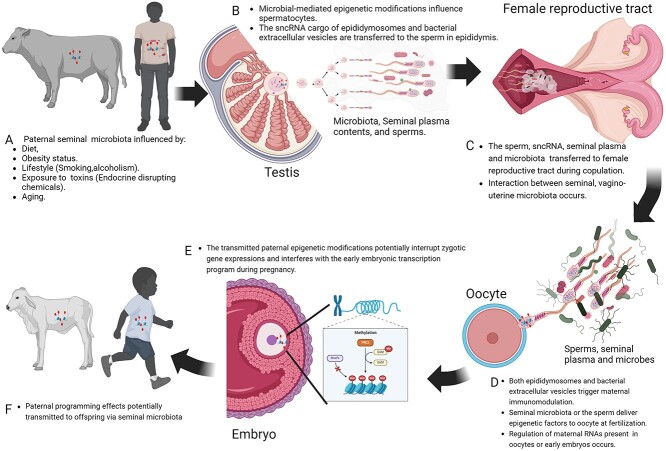
Schematic demonstration of the potential involvement of the seminal microbiota in paternal programming: The seminal microbiota could be influenced by several paternal factors like diet, obesity, stress, lifestyle choices (smoking, alcohol consumption, etc.), exposure to toxins, and aging (A). The seminal microbiota potentially detects paternal these environmental stressors and generate signaling molecules that affect their endocrine, immune, nervous systems and the overall testicular microenvironment. These signals could also influence the gene expression profiles of their developing sperms. The presence of microbiota could also mediate epigenetic modifications by determining the availability of substrates for epigenetic changes, modulating the activities of epigenetic-modifying enzymes, and activating host-cell intrinsic processes directing epigenetic pathways in spermatocytes. After spermatogenesis, the sncRNA cargo of epididymosomes and bacterial extracellular vesicles are also likely transferred to the spermatozoa (B). During copulation, the sperm, sncRNA cargo, and the seminal microbiota are deposited into the female reproductive tract, where interactions between semino-vagino-uterine microbiota occur (C), and the paternal epigenetic factors are potentially delivered to the oocyte at fertilisation. Epididymosomes and bacterial vesicles could also trigger maternal immunomodulation whereby immune cells (e.g. Polymorphonuclear leukocytes) regulate uterine endometrial inflammation and facilitate embryo implantation and contribute to post-conception fetal development (D). These paternal epigenetic modifications potentially disrupt zygotic gene expressions and influence early the embryonic transcription processes during pregnancy (E). Ultimately, the seminal microbiota-mediated paternal programming effects are potentially transmitted to the offspring (F). Figure created using BioRender.com.

While the exact mechanisms of how the seminal microbiome transfers paternal programming effects to the resulting offspring is largely not understood, our insights from the current microbiome research have enabled us to propose potential mechanisms by which the seminal microbiota could contribute to paternal programming as illustrated in a schematic diagram Figure 2. The first potential mechanism of microbial-mediated transfer of paternal programming effects to offspring may involve the influence of the seminal microbiota on sperm epigenetic modifications during the initial stages of spermatogenesis, progressing through sperm transportation along the male genital tract. Here the potential presence of microbiota in the testicular and accessory components could possibly affect the availability of substrates for epigenetic changes, modulates the activities of epigenetic-modifying enzymes, and activates host-cell intrinsic processes directing epigenetic pathways [212]. According to a recent report, certain aspects of paternal programming may begin in the testicles, epididymis, and accessory sex glands during the production of seminal plasma, which is incorporated into the semen shortly before ejaculation [1, 14, 121, 123]. During early sperm maturation, its believed that DNA methylation and histone modification occur mostly in the testicle, whereas sperm microRNA (miRNA) loading occurs in later parts of the reproductive tract. An analysis of non-coding RNAs (sncRNA) in bull sperm revealed that miRNA content of sperm begins at relatively low levels in the testicular parenchyma and increases significantly during the 14-day transit through the epididymis [123]. Moreover, the seminal microbiota differs in community structure and composition between bulls with high or poor fertility [142], just like other characteristics identified as fertility indicators, such as sperm methylation [225], miRNA abundance [226], and histone alterations [227]. Studies have shown that during fertilization, sncRNA can affect either the maternal cytoplasmic RNAs present in the oocyte, or the early embryo’s transcriptome in the embryonic nuclei [228].

Epigenetic regulation is an important mechanism by which the microbiota can affect male host physiology and can occur via several potential pathways [212]. These mechanisms include microbial biosynthesis and generation of epigenetic substrates like chemical donors for DNA or histone modifications which regulate the expression and activity of epigenetic modifying enzymes, and activate host-cell intrinsic processes associated with epigenetic pathways [212]. For example, methyl and acetyl donors are usually necessary for the enzymatic activities of histone acetyltransferases and DNA/histone methyltransferases (HATs and DNMTs/HMTs). Various commensal bacteria, notably *Bifidobacterium* and *Lactobacillus* species which are commonly used as probiotics, can metabolize one-carbon compounds into S-adenosylmethionine (SAM), which is the major substrate for DNA and histone methylation [229]. Besides, certain microbes (e.g. *Corynebacterium glutamicum, and Escherichia coli*) have the ability to convert dietary methionine into SAM [230], hence the presence and relative abundance of such bacterial species in the male reproductive system can potentially affect the availability of SAM, leading to changes in the host’s DNA or histone methylation status. Commensal bacteria also produce many SCFAs, which inhibit histone deacetylases, leading to chromatin changes associated with upregulation of gene expression [231]. The SCFAs also affect acetyl coenzyme A levels, influencing tricarboxylic acid (TCA) intermediates and ten-eleven translocation enzyme methyl cytosine dioxygenases that impact DNA methylation [232]. Research has demonstrated that SCFA-supplementation to germ-free mice may restore gut microbiota-induced transcription and epigenetic modifications [233]. All these recent evidence highlight the extensive regulation that commensal microbes especially those present in both the gut and the male reproductive tract could exert by altering host epigenetics through non-covalent modifications, histone changes, DNA methylation patterns, and SCFA-mediated immune cell regulation [212]. These epigenetic modifications mediated by the seminal microbiota could transmit paternal programming to offspring, thus influencing developmental homeostasis as well as their lifelong health and disease patterns. Considering that the seminal microbiota resides in the same anatomical region as sperm cells, it is reasonable to speculate that its influence on epigenetic alterations in sperm cells, could ultimately affect offspring health outcomes [234–238]. Hence a potential mechanism through which the seminal microbiota transmits paternal programming effects is by influencing sperm epigenetics and ultimately offspring epigenetics at conception and during early embryonic development. These influences are likely associated with changes in the methylome and transcriptome across various organs, involving early genetic modifications in the fetal organs that ultimately result into permanent phenotypic changes [239].

The second potential mechanism of transferring paternal programming effects to the offspring via seminal microbiota could be mediated via the interaction between epididymosomes and bacteria-derived extracellular vesicles (bEV) present in the semen (Figure 2). The bEVs could carry miRNAs and sncRNAs into the spermatozoa during the sperm’s transit through the epididymis [240]. Epididymosomes are a diverse array of small EVs (50–250 nm in size) generated by epididymal epithelial cells [241]. Epididymosomes, originating from the epididymal epithelium, encompass proteins, noncoding RNAs, and specific lipids. These constituents are conveyed to spermatozoa during their transit through various epididymal regions, contributing to sperm maturation and enhancing fertilizing capabilities [240]. Some bacteria are also known to generate nano-sized particles containing biomolecules such as outer-membrane proteins and lipopolysaccharides (LPS) which are also considered as bEV [242]. Briefly, both Gram-positive and Gram-negative bacteria produce bEVs from their parental bacteria, which contain a plethora of constituents, including proteins, lipids, lipoproteins, DNA, and RNA from their parental bacteria [242]. The bEVs exhibit diverse synthetic pathways and cargo-sorting mechanisms, leading to the generation of different bEV subtypes with varied cargo compositions and likely diverse biological functions reviewed in [243–245]. Typically, EVs produced by Gram-positive bacteria are termed cytoplasmic membrane vesicles, while those originating from Gram-negative bacteria are labeled as outer-membrane vesicles due to their derivation from the outer membrane [242]. EVs play crucial roles in diverse physiological functions, including the efficient clearance of redundant cellular constituents, aiding in cell maturation and adaptation to changing surroundings, and initiating coagulation processes, reviewed in [246]. Furthermore, they regulate the functions of neighboring cells by relaying intercellular signals [247]. Serving as messengers, EVs influence the functions of nearby cells through their surface proteins, enclosed cargo (such as proteins and RNAs), lipids, and glycans [246]. Both cytokines and EVs act as intermediaries in intercellular communication, with cytokines having the ability to associate with EVs either internally or externally [248–250]. These bEV-associated molecules could activate cell-surface and intracellular receptors of endometrial cells that initiate innate immune signaling, leading to immunomodulation via inflammatory pathways [242, 244, 251]. Moreover, a recent study showed that maternal microbiota-produced extracellular vesicles could act as a vital intermediary in the interaction between the maternal microbiota and the developing fetus, potentially serving as a critical factor in preparing the fetal immune system for colonization of the gut after birth [252, 253]. It is therefore possible that seminal microbiota interact with female reproductive tract microbiota and collectively transfer paternal influence on the offspring. Despite differing origins and specific functions, both bEVs and epididymosomes are transfer biomolecules that modulate the host signaling pathways and cellular functions via intercellular communication and biomolecule transfer, reviewed in [254]. Emerging evidences also indicate that sncRNAs conveyed through epididymosomes may not only impact sperm function but also play a crucial role in embryonic development and determining the health of offspring [255]. Therefore, there is a possibility that bEVs interact with host epididymosomes and bind to the spermatozoa via the receptor-ligand [256] or lipid raft–mediated cargo transfer [240] during sperm transit through epididymis. At fertilization, the cargo of epididymosomes in the sperm like sncRNAs could enter the oocyte where it potentially interferes with the stability and translational efficiency of maternal transcripts [257]. While in the zygote, these sperm-derived sncRNAs could also influence regulatory mechanisms governing early embryonic development by modifying genetic and epigenetic information that determines the health outcomes of offspring [255, 257]. It is thus possible that the bEVs could contribute to facilitating the transmission of acquired paternal traits by either activating or inhibiting transcriptional start sites, thereby regulating gene expression products in early embryos. In addition, several studies have demonstrated that male reproductive tract-derived EVs are conserved and diversified population that carry a sophisticated payload of regulatory components crucial for sperm function and may affect the biology of the female reproductive system after mating [79, 258]. In various mammalian species including mice, humans, and cattle, epididymosomes have been found to contain specific proteins, including clusterin, macrophage migration inhibitory factor, members of the cysteine-rich secretory protein family, as well as a disintegrin metalloprotease and protein disulfide-isomerase A families [259–261], which are essential for reproductive function including inflammatory mediation. From bovine epididymal fluid, two separate epididymosome populations with varied sizes, protein compositions and functions were recovered using different centrifugation techniques [262]. These two epididymosomes include the epididymal sperm binding protein 1 population that guide proteins to passing sperm through membrane fusion, as well as CD9 and other tetraspanin partners that protect sperm from reactive oxygen species [240, 261, 262]. Moreover, changes in these epididymosome components could have long-term consequences on the offspring health because the cargo carried to spermatozoa is connected to embryonic development [263]. For instance, an experiment that involved injection of miRNA from stressed males into unstressed zygotes indicated that epididymosomes contribute to offspring programming, resulting in a stressed phenotype [264–266]. It has been reported that there are distinct miRNA expression profiles seen in the gut samples of mice colonized with microbes and that of germ-free mice [267]. An inverse relationship between the abundances of miRNA and microbial perturbations have also been documented [268]. Because miRNAs are well known to regulate genes post-transcriptionally reviewed by [269, 270], it is possible that differences in the seminal microbiota could affect the expression of miRNAs in sperm and that could eventually be transferred to the progeny.

The third potential mechanism of seminal microbiome-mediated transmission of paternal programming effects to offspring could be associated with the roles of microbes in production of signaling molecules during paternal exposure to stresses or toxic substances. Several factors like diet, obesity, stress, environmental toxin exposure [271], and lifestyle choices (e.g. Smoking, alcohol consumption), psychological stress, disruptions in circadian rhythms, sleep deprivation, exposure to environmental extremes like high altitude and temperature variations, encounters with environmental pathogens, toxicants, pollutants, and noise, physical activity, and dietary factors including nutrient composition and food restriction [271] can influence the semen composition including its microbial structure [272], which may indirectly impact sperm formation and functions leading to paternal programming effects in the offspring [155, 156]. There are reports that various stress conditions during military training such as psychological stress, circadian disruption/sleep restriction, high-altitude exposure, traveler’s diarrhea, and physical activity affected gut microbiota composition and metabolome [271], therefore it is plausible that the various stress factors could potentially affect seminal microbiota structure and metabolites. It is possible that the seminal microbiota could function as paternal environmental stress detectors which then generate signaling molecules such as serotonin, g-aminobutyric acid [273], and dopamine [274], nitric oxide, carbon monoxide, hydrogen sulfide, and sulfur dioxide [275]. These molecules impact the endocrine system, immune system, nervous systems and the testis microenvironment [276]. These could influence the gene expression profiles of the developing sperm which eventually get carried to the offspring. Studies in mice have shown that exposure to tobacco smoke causes increased incidence of mutations in the germ-line heritable DNA sequence of spermatogonial stem cells, suggesting a mechanism by which paternal smoking could program gene expression in the offspring [277]. Moreover, smoking has also been associated with disturbing the balance of the gut microbiota through the similar harmful mechanisms affecting host cells [278]. Normal gut microbiota aids male reproduction via nutrition, immunity, and signaling [276]. Moreover, it has been reported that healthy gut microbiota enhances the blood-testis barrier by enhancing androgen secretion and upregulating Claudin3 protein expression [279]. There are recent research reports connecting the genitourinary microbiome, oxidative stress, DNA damage, and male fertility [280]. For example, a retrospective study involving 770 men seeking fertility assistance in the UK showed that men with *Ureaplasma* spp. and *Gardnerella vaginalis* in urine, along with *Enterococcus* spp. in semen, exhibited greater semen reactive ROS levels. The most significant ROS concentrations and elevated DNA fragmentation were observed in men with these bacteria in both urine and semen [280]. Sperm may experience oxidative stress in situations involving heat stress, obesity, hypertension, insulin resistance, changes in dietary components or nutritional patterns, and various psychological disorders [128]. These stressors could potentially induce disruptions of mitochondrial and enzymatic pathways in sperm cells resulting into oxidative stress and subsequent DNA damage [133]. Therefore, when a damaged sperm fertilizes an egg, the synergistic impacts of oxidative stress and DNA damage may trigger profound paternal programming effects on the offspring [1, 12, 133]. On the other hand, it has been reported that orally administered *Lactobacillus rhamnosus* JB1 alleviates stress-induced anxiety or depression, with its anti-stress effect mediated through the vagus nerve, as evidenced by the absence of this effect in vagotomized mice [281]. This evidence could also suggests that dysbiosis of the seminal microbiota, which typically occurs after antibiotic treatments, may be connected to DNA damage and oxidative stress in sperm. Therefore, it is possible that the seminal microbiota acts as a sensor to various paternal stress factors such as diet, obesity, environmental toxin exposure, lifestyle choices and antibiotic therapy which influences their structure, functions [282–284] and eventually transmit these effects to their offspring through either direct or indirect microbial mediated processes. These associations could have significant implications for both achieving pregnancy and the subsequent health of offspring in both humans and animal species. In summary, emerging evidence suggests that the seminal microbiota potentially contributes to paternal programming through complex mechanisms including microbial-mediated epigenetic modifications during spermatogenesis, interactions between epididymosomes and bacterial extracellular vesicles during sperm transit through epididymis, and production of signaling molecules in response to paternal environmental factors such as diet, obesity, lifestyle choices (e.g. smoking, alcoholism, etc.), and antibiotic treatments which affect male reproductive health.

## Significance and future research directions

The microbiome associated with the male reproductive tract, particularly seminal microbiome may significantly influence offspring health through paternal programming of embryonic and fetal development. Hence, understanding the role of the seminal microbiome in shaping offspring health presents an important novel avenue for holistic understanding of the developmental programming in humans and animals. Future research should therefore prioritize investigating the functional features of the seminal microbiome, its seeding sources, its impact on the sperm cell development, as well as its interactions with vagino-uterine microbiome and the female reproductive immune systems. A deeper understanding these concepts could help in uncovering the role of the seminal microbiome in paternal programming of offspring health especially through elucidating the molecular mechanisms underlying seminal microbial-mediated epigenetic changes and its ultimate involvement in transfer of paternal influence to offspring. This exploration holds promise for diagnosing and treating abnormal transgenerational microbial-mediated epigenetic influences. In addition, further studies are also needed to characterize the cargo of epididymosomes and bEVs and their interactive effects on sperm function and embryonic development and long-term offspring health. Moreover, the clear impact of paternal environmental factors (such as diet, lifestyle, obesity status, toxin exposures, and stress) on seminal microbiota composition and signaling molecule production remains less understood. Given the recent hypothesis suggesting that there is a heightened impact on offspring metabolic health when sperm and seminal plasma originate from divergent dietary backgrounds compared to when they share the same dietary background [14], further research is necessary to examine the association between seminal plasma-mediated programming of the embryo and uterus and relative abundance of the seminal bacterial taxa. By addressing these questions, we can gain insights into the complex interplay between the seminal microbiome and paternal programming, with implications for both reproductive health and offspring well-being. These studies can be particularly relevant to assisted reproductive techniques in both human and animal reproduction, particularly where embryos develop without seminal plasma and are transferred to a non-seminal plasma-primed uterus. Furthermore, understanding how dysbiosis in the paternal seminal microbiome, particularly in conjunction with maternal reproductive microbiome, influences fetal immune programming and offspring health outcomes is crucial. Addressing this challenge may involve longitudinal studies tracking microbiome dynamics and health outcomes across multiple generations. Additionally, identifying biomarkers associated with dysbiosis of seminal microbiota and adverse offspring health outcomes will be essential for early diagnosis reproductive disorders and development of microbiome-targeted interventions.

One limitation in this area of research is the complexity of the interactions between the seminal microbiota, paternal factors, sperm epigenetic regulation and how the immune microenvironment of the female reproductive tract is modulated by the seminal microbiome at conception and throughout gestation. Overcoming this challenge will require interdisciplinary collaboration and the development of advanced methodologies for analyzing microbiome composition and epigenetic modifications in sperm. This will require large-scale epidemiological studies and the integration of multi-omics data to comprehensively assess microbiome-host interactions and their consequences for offspring health. In summary, future research should focus on unravelling the mechanisms linking the seminal microbiome to paternal programming of offspring health and addressing challenges regarding the microbiome complexity and its related biomarkers through interdisciplinary collaboration and advanced methodologies. This will facilitate the development of microbial-targeted interventions to mitigate adverse offspring health outcomes.

## Conclusions

Although the concept of paternal programming is still in its infancy, the emerging body of evidence suggests that the seminal microbiome through its interaction with the vagino-uterine microbiome may play a pivotal role in shaping offspring health by paternal programming of embryonic and fetal development. In livestock production, exploration of the seminal microbiome as a factor in paternal programming of offspring health represents a paradigm shift and may lead to the development of seminal microbiome-targetted innovative breeding strategies and management practices that optimize offspring health, enhance genetic selection, and improve overall herd productivity. In humans, understanding the contribution of the seminal microbiome to paternal programming has the potential to improve both parental reproductive health and child health outcomes by personalizing prebiotic/ probiotic therapies to promote beneficial bacteria, modulate the immune system, and suppress harmful microbiota in fathers during preconception period. As more research continues to unfold, collaboration between geneticists, microbiologists, physiologists, and livestock experts will be essential in harnessing the potential of the seminal microbiome to drive positive changes in both humans and livestock production. Further investigations into how the seminal microbiome influences conception, maternal immune systems, fetal development as well as the resulting offspring health outcomes is a promising research avenue.

## Author contribution

JK and SA conceptualized and outlined the review, performed the literature search, and drafted the initial manuscript. JK, CD, LR, and SA were involved in manuscript writing, revision, editing, and finalization. All authors contributed to the article and gave their approval for the submitted version.

## Funding information

The financial support for the work presented in this review was partially provided by the North Dakota Agricultural Experiment Station as part of a start-up package for SA.

## Data availability

The sequencing data for the V3-V4 region of the 16S rRNA gene used in this study are available upon request.
